# End-to-end antigenic variant generation for H1N1 influenza HA protein using sequence to sequence models

**DOI:** 10.1371/journal.pone.0266198

**Published:** 2022-03-28

**Authors:** Mohamed Elsayed Abbas, Zhu Chengzhang, Ahmed Fathalla, Yalong Xiao

**Affiliations:** 1 School of Computer Science and Engineering, Central South University, Changsha, China; 2 The College of Literature and Journalism, Central South University, Changsha, China; 3 Mobile Health Ministry of Education-China Mobile Joint Laboratory, Changsha, China; 4 Department of Mathematics, Faculty of Science,Suez Canal University, Ismailia, Egypt; Fuzhou University, CHINA

## Abstract

The growing risk of new variants of the influenza A virus is the most significant to public health. The risk imposed from new variants may have been lethal, as witnessed in the year 2009. Even though the improvement in predicting antigenicity of influenza viruses has rapidly progressed, few studies employed deep learning methodologies. The most recent literature mostly relied on classification techniques, while a model that generates the HA protein of the antigenic variant is not developed. However, the antigenic pair of influenza virus A can be determined in a laboratory setup, the process needs a tremendous amount of time and labor. Antigenic shift and drift which are caused by changes in surface protein favored the influenza A virus in evading immunity. The high frequency of the minor changes in the surface protein poses a challenge to identifying the antigenic variant of an emerging virus. These changes slow down vaccine selection and the manufacturing process. In this vein, the proposed model could help save the time and efforts exerted to identify the antigenic pair of the influenza virus. The proposed model utilized an end-to-end learning methodology relying on deep sequence-to-sequence architecture to generate the antigenic variant of a given influenza A virus using surface protein. Employing the BLEU score to evaluate the generated HA protein of the antigenic variant of influenza virus A against the actual variant, the proposed model achieved a mean accuracy of 97.57%.

## 1 Introduction

The fast-paced determination of circulating influenza strains is crucial for vaccine manufacturing. The virus lipid envelope consists of glycoproteins namely Hemagglutinin (HA) and Neuraminidase (NA). Given the changing nature of the influenza virus, it undergoes two types of changes identified as antigenic shift and antigenic drift. The antigenic shift in the influenza virus is a significant change in virus surface protein that leads to a new HA, NA, or both. This profound change happens when the virus, which affects a certain population, gains the ability to infect another population and this type of change is less frequent and unpredictable [1]. The antigenic drift in the influenza virus is related to minor changes that occur in the surface proteins of the virus: HA and NA. Moreover, these changes are more frequent especially in influenza virus A [2]. Most influenza vaccines are designed to target an influenza virus’s surface proteins.

The changes occurring in the influenza virus are primarily caused by changes in the virus envelope. These changes in the outer surface of the influenza virus account for the virus evasion from antibodies created during prior infections [3]. Several studies were conducted to predict the virus antigenic variant based on the virus antigenic data [4–6]. The virus antigenic distance data, acquired from Hemagglutinin-Inhibition (HI) assay, calculates the antigenic distance between pairs of the influenza virus. Moreover, the antigenicity data is obtained in a laboratory setup, and this process requires a considerable amount of work and time [7].

Dramatic breakthroughs have been realized recently in deep learning. Applications such as natural language processing (NLP) [8, 9], image recognition [10, 11], and Bioinformatics [12, 13] have seen tremendous advancements due to deep learning (DL) and hardware acceleration capabilities. Motivated by these recent developments, a computational method was devised to generate HA sequence of the antigenic variant of a given influenza virus based on a hypothesized antigenicity value, utilizing both influenza antigenicity data and sequences. The proposed model relies on a framework for solving sequential problems namely sequence to sequence [14]. Sequence to sequence methodology has obtained popularity due to its high efficacy in various computational modeling tasks, especially when an attention mechanism is added [15, 16], such as machine translation [15–18], text summarization [19–21], image captioning [22–24] and video captioning [25, 26], and speech recognition [15, 27, 28]. The influenza virus outer surface proteins are represented as a sequence of letters. Considering the (HA) protein representation and advancement in (NLP) domain, the problem can be formulated as an (NLP) problem taking the antigenic distance into consideration. In this work, a variety of sequence to sequence deep learning models were implemented on pairs of influenza HA sequences, based on their antigenic relation; in an attempt to generate the antigenic pair of a newly emerging influenza virus.

## 2 Related work

Many studies were conducted to devise computational methods for specifying a new influenza virus antigenicity. Such studies employed a variety of machine learning models in an attempt to identify the antigenic pair of an influenza virus. However, the studies mentioned in upcoming sections, in general, formulated the problem such that the input is the sequence of an existing virus and the sequence of newly discovered one, and the output defines the antigenic distance between both sequences as a classification or regression problem. Additionally, these studies are mentioned even though the approach is to generate the antigenic pair based on a given antigenic distance. These machine learning methods are mainly categorized into four categories; classical, tree-based, stacking and ensemble, and deep learning methods. The following subsections elaborate more on the work done, under each of the mentioned categories.

### 2.1 Classical methods

Liao et al. [29] compared several techniques, particularly, iterative filtering, multiple regression, logistic regression, and support vector machine. These methods were applied after utilizing a grouping method for polarity, charge, and structure of amino acids on the H3N2 subtype. Moreover, the final results showed the superiority of the grouping method followed by iterative filtering. Sun et al. [30] devised a methodology based on bootstrapped ridge regression with antigenic mapping to specify influenza virus antigenicity using (HA). Their work was applied on H3N2 sequences. Peng et al. [4] introduced a universal computational model named PREDAV-FluA, built based on the regional bands, and employed a Naive Bayes classifier technique. PREDAV-FluA was applied on subtypes H1N1, H3N2, and H5N1.

### 2.2 Tree-based methods

Neher et al. [31] conducted research, based on HA sequences and phylogenetic tree, to predict the antigenic evolution of different influenza virus types. The researchers utilized two related models a tree-based model and an amino acid substitution model. The mentioned models performed similar or better than cartographic approaches. Yao et al. [32] utilized a joint random forest regression technique that relies on the choice of amino acids substitution matrices and antigenic cartography on the H3N2 subtype. Bandi et al. [33] predicted rates of circulating viruses for the upcoming influenza season using historical data by applying a tensor completion formulation. Additionally, the suggested vaccine efficiency was predicted by implementing an optimal regression trees technique using the distances between the vaccine strain and the actual circulating viruses in influenza season for H1N1, H3N2 subtypes, and influenza type B. Finally, the mentioned model scored well, and their work did not utilize the influenza HA sequence.

### 2.3 Stacking and ensemble

Yin et al. [5] made an ensemble stack of different classifiers based on each classifier strength. The first level of the stack consisted of an ensemble between three classifiers, namely logistic regression, neural network, and Naive Bayes. The second level of the stack incorporated an ensemble of random forest and gradient boosting. Finally, the third level of the stack employed logistic regression classifier. The mentioned stack of ensembles of classifier models was applied after feature extraction on the influenza H1N1 subtype. The authors compared three feature extraction methods based on regional bands, epitope regions based, and residues, where the latter method gave the best performance. The work done by Peng et al. [34] produced an ensemble of three computational steps. The first step is based on the Naive Bayes classifier which is used to predict the antigenic relation between two viruses. The second step is to connect viruses with similar antigenicity predicted from the first step. After that, a correlation network is constructed based on predicted antigenicity. Finally, the network clustering technique is applied to create antigenic clusters. The ensemble, PREDAC, as depicted by the authors is capable of predicting antigenicity and antigenic variants for influenza types A and B.

### 2.4 Deep learning

The authors of [35] used an encoder-decoder architecture of recurrent neural networks which enabled them to implement a sequence to sequence prediction on the influenza H3N2 subtype. The deep learning model employed techniques from (NLP) such as embedding and using a 3-gram residue method for each sequence step for prediction. The model performance was excellent, yet, the authors had to train the model on virus sequences in chronological order without considering the actual value of antigenic distance. Yin et al. [36] used a similar methodology used in [35] in addition to a temporal attention technique while limiting their predictions to the sites of epitope regions. Despite the fact that the model performed exceptionally well in terms of accuracy metric, the authors neglected the antigenic distance as a variable to the model input. The approach depicted in [37] employed a six-step methodology alongside with particle swarm algorithm to optimize the hyperparameters of convolutional neural network. The computational model is trained on influenza H3N2 subtype data and utilized antigenic cartography. Forghani et al. [38] used physicochemical properties of the constituent amino acids with the help of PCA as a dimensionality reduction technique, to create a sequence encoding. The obtained sequence encoding is fed to a convolutional neural network to predict the antigenic distance for the H1N1 influenza virus subtype. Yin et al. [39] devised a framework namely IAV-CNN to predict the antigenicity of influenza A viruses. The researchers applied techniques from the NLP domain for embedding namely ProtVec, along with a 2-dimensional convolutional neural networks. The IAV-CNN framework was trained on H1N1, H3N2, and H5N1 and the CNN architecture relied on squeeze-and-excitation mechanisms.

## 3 Methodology

### 3.1 Data collection and preprocessing

We incorporated two data sets in this research; antigenic data, and sequence data of influenza A subtype H1N1. Antigenic data was based on hemagglutination inhibition (HI) assay and was collected from reports of international organizations and published papers including World Health Organization (WHO), European Centre for Disease Prevention and Control (ECDC), the Francis Crick Institute (FCI), and Food and Drug Administration (FDA). Additionally, the HA protein sequences were taken from Influenza Virus Resource (IVR) [29] and Global Initiative on Sharing All Influenza Data (GISAID) [40]. The Antigenicity data consisted of three columns VirusA, VirusB, and Antigenic Distance. These columns resemble an old virus, new virus, and antigenic distance between both viruses respectively. The antigenic distance *D*_*ij*_ follows definition by Archetti-Horsfall distance [41] given by Eq 1.

$$\begin{array}{c} D_{ij}=\sqrt{\frac{H_{ii}*H_{jj}}{H_{ij}*H_{ji}}} \end{array}$$ (1)

where *D*_*ij*_ is the antigenic distance, *H*_*ij*_ is the hemagglutination inhibition titer for a given strain *i* in relation to the antisera caused by another strain *j*. If The value of the antigenic distance is greater than or equal to 4, the two viruses are said to be antigenic distinct. Otherwise, they are considered similar [29]. Moreover, antigenic distance data and the HA protein sequence data are joined together, then data is filtered to avoid repetition. As a result of filtering, records containing an antigenic distance above 185 were removed, as the antigenic distance is far between the two strains. Finally, the obtained 998 pairs of influenza virus A subtype H1N1 with sequences and their antigenic distance. A key point of the research was creating an end-to-end model without any feature extraction, or feature engineering employing the strength of deep learning models.

### 3.2 Models construction

**Fig 1:**
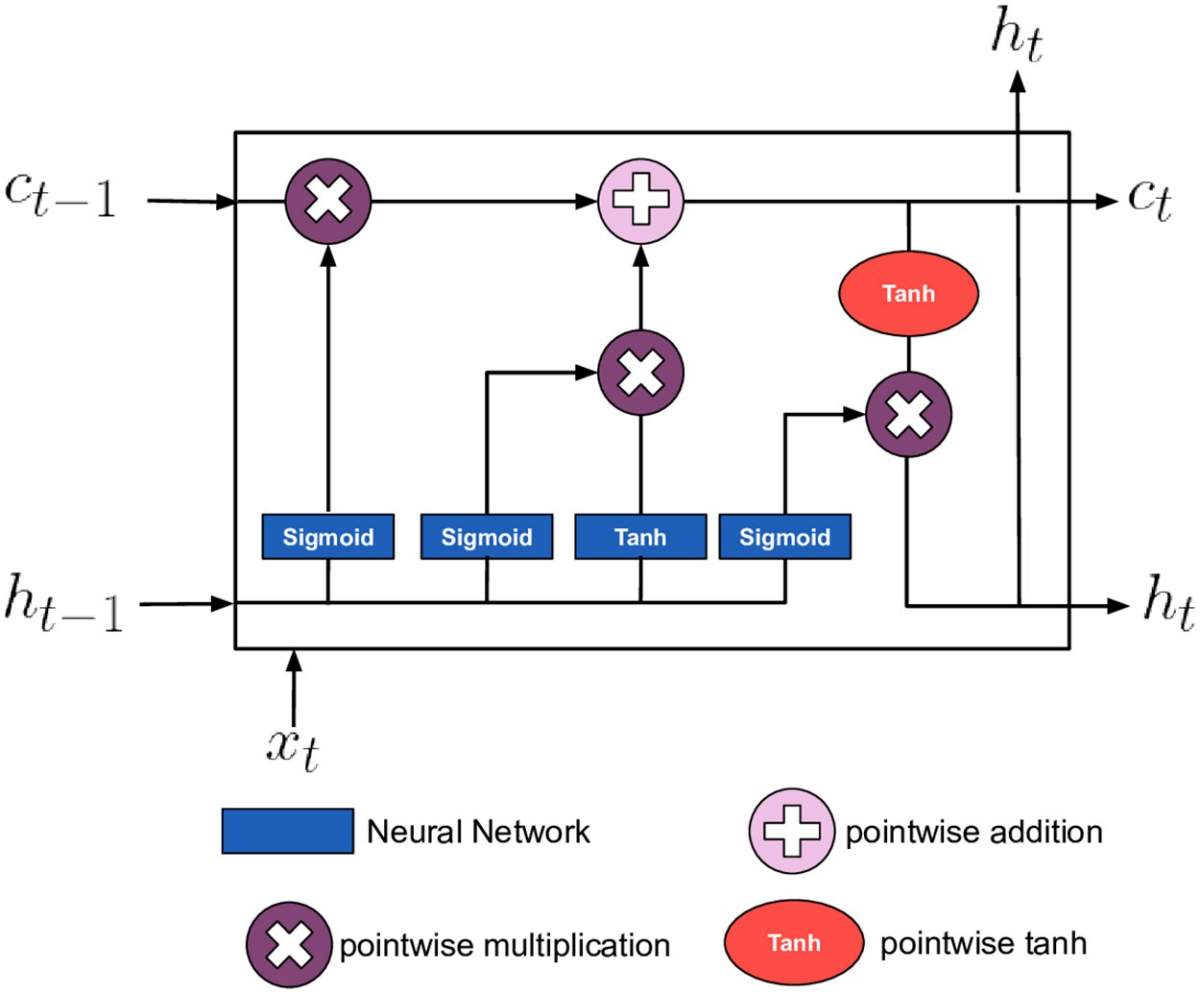
LSTM cell.

**Fig 2:**
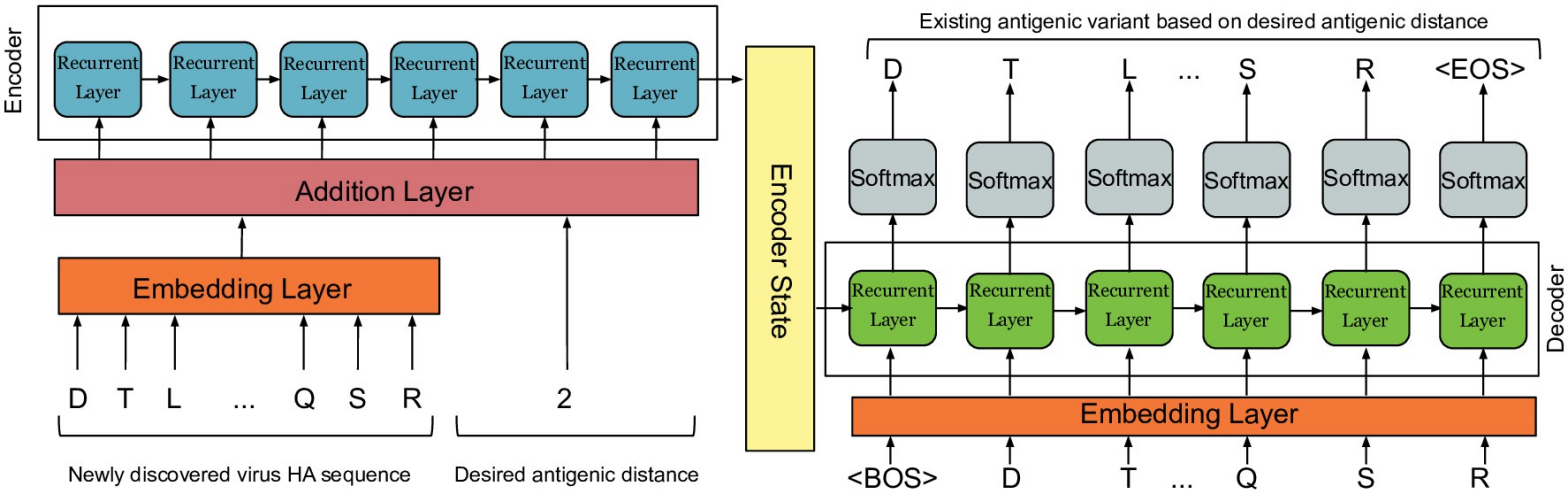
Sequence to sequence architecture.

**Sequence to sequence** model is typically an encoder-decoder architecture [14]. The basic building unit in both encoder and decoder is typically a recurrent neural network such as LSTM [42] or GRU [43] layers, which are illustrated in Fig 1 respectively. First, at each step of the sequence, the hidden state vector is computed. Then at the following step, the hidden state is computed considering the hidden state of the previous step along with the current step input until a final hidden state vector is computed. Second, the decoder receives the final hidden state vector from the encoder as an input. Then, at each step, a new hidden state vector is computed considering the hidden state of the prior cell and the current step target. Eventually, a vector containing the probabilities for all domain items is produced to represent the following item in the sequence as shown in Fig 2.

In this work, various architectures of sequence to sequence models were utilized. relying on different types of recurrent neural networks and a number of consecutive layers of the same recurrent neural network to search for the best scoring architecture. Table 1, is an illustration of the various architectures used in this research. Moreover, the inputs to sequence to sequence model are newly discovered virus sequence, antigenic distance, and old virus sequence. In training mode, a new virus sequence and antigenic distance are given to the encoder, while the old virus sequence is given to the decoder. In inference mode, a newly discovered virus sequence and a hypothesized antigenic distance are given to the encoder inference model, after that the decoder inference model is initiated with a beginning of a sentence marker to start predicting the old virus sequence, based on the hypothesized antigenic distance. Finally, data for both antigenic distinct and similar sequences were utilized. However, the main interest was obtaining a known virus with high antigenic similarity to discovered virus i.e. antigenic distance between both viruses is less than 4, data for both antigenic distinct and similar viruses were used. The developed models are anticipated to aid in the development of vaccines by eliminating the necessity for Hemagglutinin-Inhibition (HI) assay once the newly found virus sequences are identified.

**Table 1:** Utilized models’ architectures.

Model name	Encoder		Decoder		Attention layer
	Recurrent layer type	No. of recurrent layers	Recurrent layer type	No. of recurrent layers	
LSTM	LSTM	1	LSTM	1	No
GRU	GRU	1	GRU	1	No
Deep_LSTM	LSTM	2	LSTM	1	No
Deep_BI_LSTM	BI_LSTM	2	LSTM	1	No
Deep_GRU	GRU	2	GRU	1	No
Deep_BI_GRU	BI_GRU	2	GRU	1	No
Attn_Deep_BI_LSTM	BI_LSTM	2	LSTM	1	Yes
Attn_Deep_BI_GRU	BI_GRU	2	GRU	1	Yes

**Fig 3:**
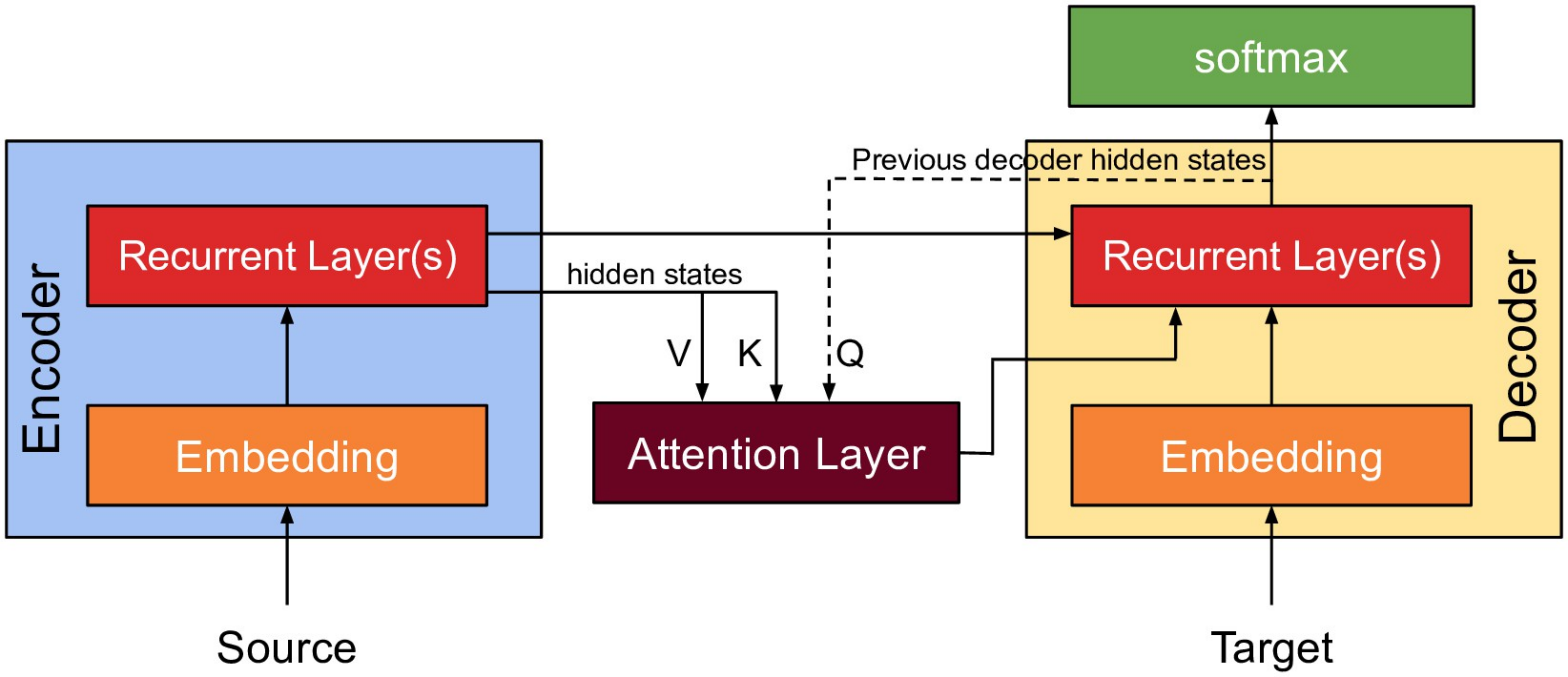
Attention with sequence to sequence architecture.

The nature of sequence to sequence models depends on the final hidden state of the encoder; and in the case of lengthy sequences, there is a significant probability that the initial context has been lost by the end of the sequence. **Attention mechanism** combats losing context in long sequences by accurately encoding the parts of the input sequence that surround a particular word, or in this case amino acid. In this research, the attention mechanism used in [15] was utilized with the sequence to sequence model, as illustrated in Fig 3. The model consists of an encoder, a decoder, and an attention layer. Firstly, the encoder produces hidden states of each element in the input sequence. Secondly, the attention layer computes alignment scores using the previous decoder hidden state as query and the encoder hidden states as both keys and values. After that, the resulting alignment scores are incorporated in a single vector, then a softmax layer is applied. Finally, the resulting alignment scores and their respective hidden states are multiplied to form a context vector. Consequently, the previous decoder output and the resulting context vector are concatenated and given to the decoder accompanied by the previous decoder’s hidden state.

The acquired sequences data represent the HA protein of an influenza subtype H1N1. Each sequence consists of 327 letters representing different amino acids. In resemblance to an NLP problem, considering each letter as a word and the whole sequence as a sentence. Therefore, word **embedding** methodology [44] is employed to represent each sequence in a comprehensible way for a computational method. In this implementation, an embedding size of 50, batch size of 128 were used. Consequently, each sequence is represented as a tensor of shape (128, 327, 50). **learning rate decay** is not only recommended as depicted in [45], it is also considered a significantly important technique while training neural networks [46]. Thus, the step decay technique is employed to lower the initial learning rate with a decay factor of 0.98 and a drop rate (step size) of 50 iterations. The step decay learning rate is given by Eq 2.

$$\begin{array}{c} \alpha_{n}=\alpha_{0}*d^{floor(\frac{1+n}{r})} \end{array}$$ (2)

where *α*_*n*_ is the learning rate at epoch, *α*_0_ is the initial learning rate, *d* is the decay parameter, and *r* is the drop rate.

## 4 Experiments and results

### 4.1 Baseline architectures

Two different architectures were used as shown in Table 1 as the baseline models, namely LSTM and GRU. Both architectures have a single recurrent layer for decoder and encoder, and the recurrent layers used are LSTM and GRU as depicted by models names. The sole objective for using the two architectures as baseline models is to compare deeper models performance against these shallow architectures.

### 4.2 Experimental setup and implementation

Architectures in Table 1 were executed using the parameters stated in Table 2. The number of epochs is set to 500 with a patience of 11. Thus, the model in training mode will only stop if the training accuracy did not improve for 11 epochs. Recurrent layers of both encoder and decoder use a dropout rate of 0.2. It is commonly agreed that using dropout will regularize layers learning and prevent overfitting. Each model will randomly select 80% of the data for training and the remaining 20% for test. Additionally, the training data is split into 70% for training and 30% for validation. Each of the proposed architectures is executed in training mode until reaching the early stopping threshold. learning rate will decay every 50 epochs with a decay factor of .98, as slowing the learning rate will enhance the learning of complicated patterns and is commonly believed to help neural networks find a local minimum. After that, each model is evaluated by performing predictions on the test data set. Finally, generated and actual sequences are saved for test evaluation and architectures comparison. The work done in this research is implemented in the Python programming language. Libraries Such as Biobython for reading sequence data and Keras were used for developing the proposed deep learning architecture. Full implementation and data are made available on GitHub.

**Table 2:** Proposed architectures parameters.

Parameter	Value
Epochs	500
Batch size	128
Early stopping patience	11
No. of recurrent layer cells	50
Learning rate	0.001
Decay step size	50
Decay Factor	0.98
Dropout rate	0.2

### 4.3 Accuracy metrics

As a final step, the generated sequences acquired during the test phase are evaluated against the original sequences. Since the approach taken in this paper is similar to approaches used in NLP, it was convenient to use accuracy metrics used in NLP as well. Hence, the utilization of the BLEU score [47] which is often used in NLP problems. BLEU score consists of two main terms, particularly precision score and a brevity penalty. The precision score, *p*_*n*_, calculated for each n-gram length by summing over the match for a generated sequence *S* against the original sequence *O* from the test set as in Eq 3.

$$\begin{array}{c} p_{n}=\frac{\sum_{S\in O}\sum_{n-gram\in S}Count_{matched}\left(n-gram\right)}{\sum_{S\in O}\sum_{n-gram\in S}Count\left(n-gram\right)} \end{array}$$ (3)

Since the BLEU score is precision-based, and recall term will be inconvenient to formulate over multiple references, thus, the brevity penalty calculated in Eq 4 is introduced to rectify the possibility of presenting high precision hypothesis.

$$\begin{array}{c} BP=\{\begin{array}{ccc} 1 & if & L>r\\ e^{1-r/L} & if & L\leq r \end{array} \end{array}$$ (4)

where *L* is the output sequence length and *r* is reference sequence length.

$$\begin{array}{c} BLEU=BP*exp(\sum_{n=1}^{N}w_{n}logp_{n}) \end{array}$$ (5)

where *w*_*n*_ represents the weights and typically equals 1/*N* and *N* represents the number of grams. According to the definition given by Eq 5 BLEU score will always range from 0 to 1. Thus, for each generated sequence compared against the original sequence, the resulting BLEU score will represent the accuracy of the model in generating a single sequence. Finally, the mean BLEU score percentage was used as an indicator of each model’s accuracy.

### 4.4 Results

During the training stage, architectures utilizing bi-directional recurrent layers—both LSTM and GRU—performed better than other architectures in terms of both training and validation accuracy, as shown in Table 3. Unexpectedly, the addition of an attention layer to the encoder-decoder architecture did not improve training or validation accuracy. Moreover, in the case of using bi-directional LSTM recurrent layer in encoder and decoder with additional attention layer, both the training and validation accuracy were lower than the chosen baseline architectures.

**Table 3:** Training performance of the proposed models.

Model	Training		Validation		Epochs
	Loss	Accuracy	Loss	Accuracy	
Deep_BI_GRU	0.0581	0.9801	0.0576	0.9814	338
Deep_BI_LSTM	0.0684	0.9801	0.0619	0.9818	445
Attn_Deep_BI_GRU	0.1094	0.9685	0.1060	0.9682	143
Deep_LSTM	0.1400	0.9652	0.1205	0.9687	488
Deep_GRU	0.1578	0.9588	0.1320	0.9651	258
LSTM	0.1968	0.9577	0.2120	0.9496	314
GRU	0.1928	0.9519	0.1617	0.9555	187
Attn_Deep_BI_LSTM	0.2602	0.9476	0.3100	0.9325	113

**Fig 4:**
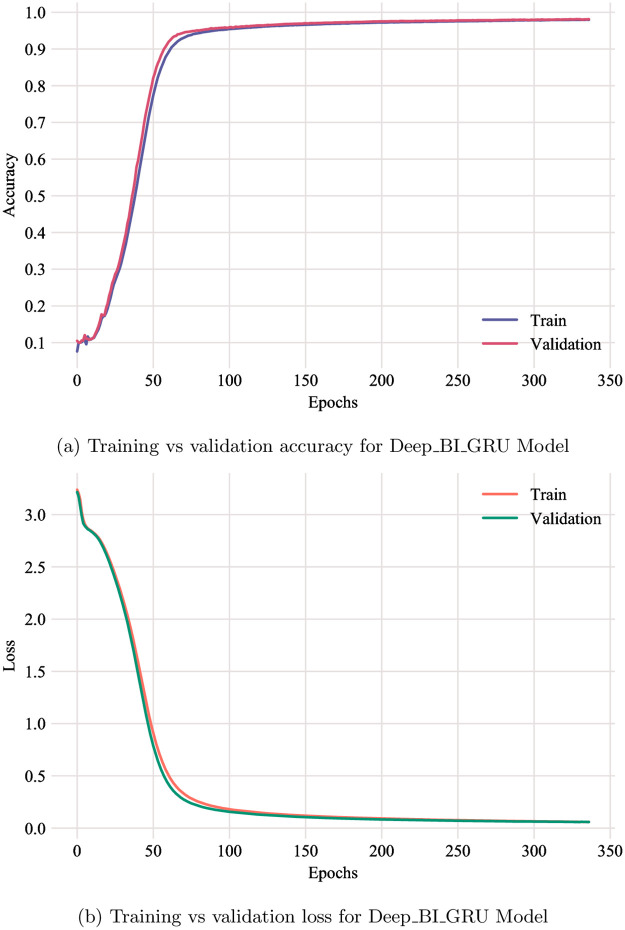
Training vs validation. (a) Training vs validation accuracy for Deep_BI_GRU Model. (b) Training vs validation loss for Deep_BI_GRU Model.

Fig 4 shows the improvement of training accuracy and loss compared to the validation over epochs for the architecture utilizing bi-directional GRU as a recurrent layer. However, the improvement in accuracy was very small after approximately 60 epochs. It is notable that, the loss and accuracy of both training and validation are almost identical, with a very small generalization gap. Moreover, both loss and accuracy reach a point of stability at the end of training epochs. This typically indicates that the model is a good fit.

Table 4 illustrates proposed architectures ordered by mean accuracy for 1-gram, 2-gram, 3-gram, and 4-gram BLEU score on the test set. Surprisingly, the best-performing architecture was the one based on bi-directional GRU layers, despite the expectations that the introduction of attention would improve accuracy. Nevertheless, the bi-directional LSTM with attention and deep GRU performed less than baseline architectures.

**Table 4:** *n*-gram BLEU accuracy (%) of the proposed models.

Model	Mean accuracy			
	BLEU-1	BLEU-2	BLEU-3	BLEU-4
Deep_BI_GRU	97.57%	91.44%	86.02%	82.70%
Deep_LSTM	97.28%	90.44%	84.46%	80.95%
Deep_BI_LSTM	97.14%	89.57%	83.07%	79.01%
Attn_Deep_BI_GRU	96.36%	85.49%	75.42%	70.71%
LSTM	95.74%	82.61%	69.23%	63.19%
Deep_GRU	95.45%	81.66%	67.51%	61.29%
Attn_Deep_BI_LSTM	94.92%	81.10%	67.41%	60.84%
GRU	90.21%	75.17%	60.61%	54.33%

**Fig 5:**
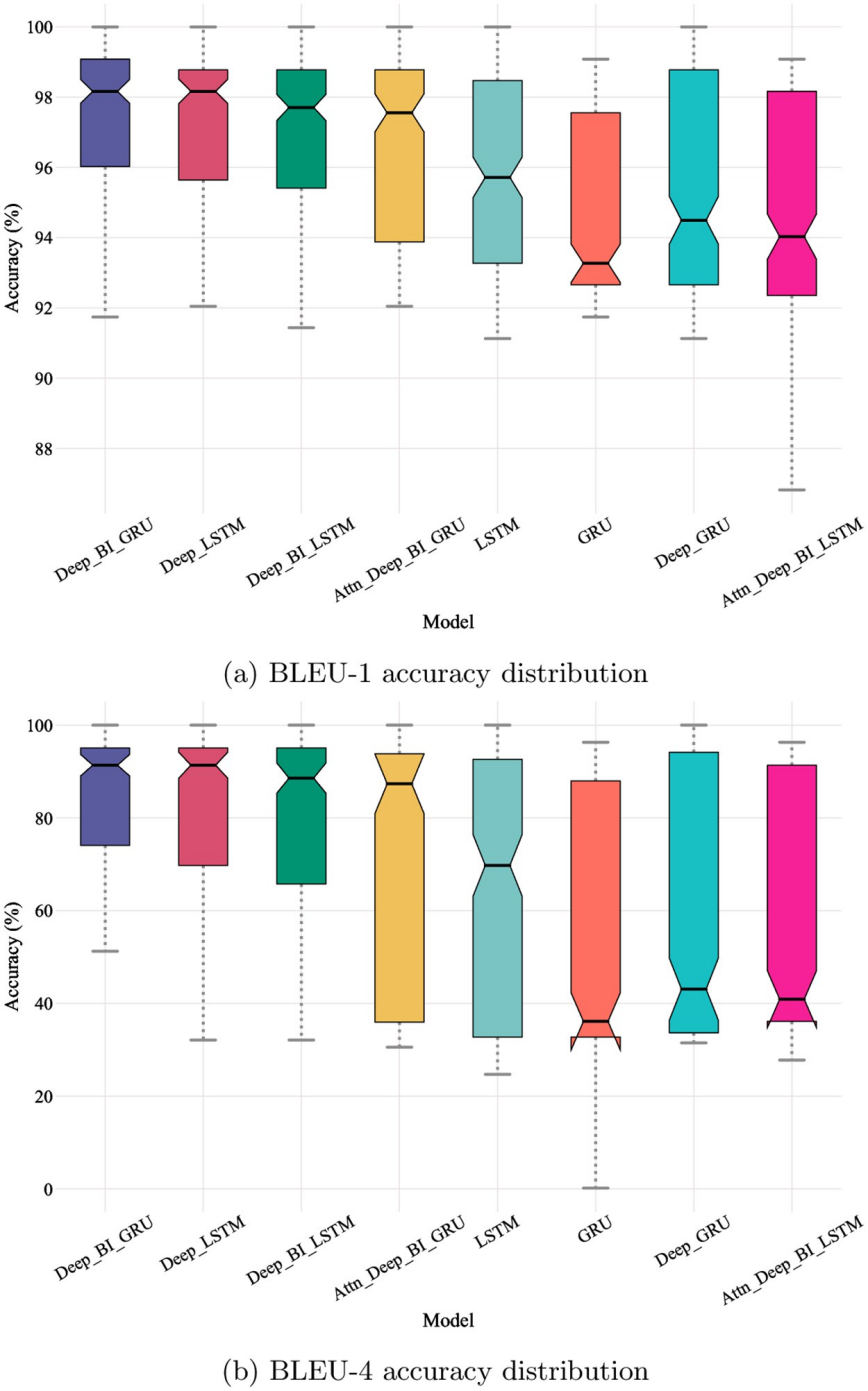
Accuracy distribution. (a) BLEU-1 accuracy distribution. (b) BLEU-4 accuracy distribution.

Additionally, the bi-directional GRU architecture achieved a higher mean accuracy. However, the gap between this architecture and attention-based architectures is fairly high, which indicates that attention might not be helpful in this case. Fig 5 shows box plots of results distribution for each architecture for 1-gram and 4-gram BLEU scores. The leading architecture (Deep_BI_GRU), when evaluated with a 1-gram BLEU score shows a maximum accuracy of 100% and a median accuracy of 98.5% approximately where the first quartile is above 96%. In other words, generated sequences accuracy distribution is right-skewed with a minimum accuracy of 92%, which indicates a high quality in generated sequences when compared to original sequences. On the other hand, the LSTM architecture shows more diversity in results with a lower mean accuracy of 96% approximately and a minimum accuracy below 92%. Finally, the bi-directional LSTM with attention model shows even higher variations in generated sequences quality where the minimum falls below 88%. clearly proposed architectures have consistent accuracy distributions over 4-gram BLEU scores where the bi-directional GRU architecture is explicitly performing better than other architectures.

### 4.5 Discussion

The fast-paced development of sequencing methods has made influenza protein sequencing a standard segment of the influenza research process and vaccine determination. The recently discovered influenza protein sequences are saved in influenza databases like the NCBI influenza virus resource (IVR) database. If effective sequence-based antigenic prediction tools can be created, much of the burden of influenza monitoring may be alleviated. This work highlighted the process of generating an antigenic variant for influenza H1N1 viruses with little antigenic data. Moreover, a model based on additional antigenic data would better reflect the overall structure behind antigenic variation. To the best of authors knowledge, and at the time of writing this paper, there is no study in the literature that uses a generative modeling technique to address the problem of the influenza virus antigenicity.

In this work, a single-step methodology was adopted that does not require feature engineering, and it is not dependent on virus discovery chronological order in training or prediction. Hence, this methodology requires less computational effort. Furthermore, integrating this methodology into existing influenza virus databases will alleviate a colossal part of antigenic pair identification and vaccine design.

Finally, the metric utilized in this work namely BLEU is generally accepted in NLP problems. Since there is no conventional computational method for measuring the quality of generated sequences, the BLEU score is seen as an effective metric in measuring the quality of generated sequences.

## 5 Conclusion

Throughout this work, antigenicity data was used along with influenza H1N1 HA sequences to generate the antigenic pair of influenza viruses. Exploiting the advancement in deep learning to evaluate different architectures to find the best suitable architecture for the task at hand. The main gain in this research is obtaining an end-to-end model avoiding feature engineering, utilizing a single-step prediction, and evading chronological order training. This work opens a new door towards generating the antigenic pair of an influenza virus depending on the antigenic distance in a novel way. This work can help to reduce the time consumed to define the antigenicity of a newly discovered H1N1 influenza virus relying on the virus HA sequence and antigenic distance. Furthermore, this work can accelerate vaccine selection and manufacturing. As demonstrated in this search generative modeling techniques are viable for addressing the influenza subtype H1N1 antigenicity problem. For future work, the authors are planning to experiment with different generative methodologies to generate the antigenic variant for influenza type A viruses and attempt to devise a general method for all different subtypes.
